# Growing threat of emerging and reemerging diseases: arboviruses and vector-borne diseases in the Americas

**DOI:** 10.17843/rpmesp.2024.411.13805

**Published:** 2024-03-27

**Authors:** César Cabezas, Pedro F. C. Vasconcelos

**Affiliations:** 1 Instituto Nacional de Salud, Lima, Peru. Instituto Nacional de Salud Lima Peru; 2 Facultad de Medicina, Universidad Nacional Mayor de San Marcos, Lima, Peru. Universidad Nacional Mayor de San Marcos Facultad de Medicina Universidad Nacional Mayor de San Marcos Lima Peru; 3 Departamento de Patologia, Universidade do Estado do Pará, Belém, Psará, Brasil. Universidade do Estado do Pará Departamento de Patologia Universidade do Estado do Pará Belém Psará Brazil; 4 Seção de Arbovirologia e Febres Hemorrágicas, Instituto Evandro Chagas, Ananindeua, Pará, Brasil. Seção de Arbovirologia e Febres Hemorrágicas Instituto Evandro Chagas Ananindeua Pará Brasil

In recent decades, the Americas have experienced an alarming and unprecedented increase in diseases caused by arboviruses, highlighting the critical importance of addressing these challenges in the public health field. Arboviruses, an acronym for arthropod-borne viruses, are viral agents transmitted by vectors, and they have demonstrated a concerning capability to unleash devastating epidemics, affecting entire communities and posing a threat to the global health of our countries and the Region.

Dengue, Zika, chikungunya, and urban yellow fever are diseases transmitted by mosquitoes of the genus Aedes, especially *Aedes aegypti*, which have emerged as protagonists in the Americas region. In the case of dengue, as of epidemiological week 8 of 2024, a total of 1,874,021 suspected cases were reported in the Americas, of which 658,215 cases (35%) were laboratory-confirmed and 1670 (0.1%) were classified as severe dengue, resulting in a total of 422 deaths from dengue, with a lethality rate of 0.023% (cumulative incidence of 205 cases per 100,000 inhabitants). This figure represents a 249% increase compared to the same period in 2023 and a 354% increase compared to the average of the last 5 years ^1^.

Regarding chikungunya, as of week 30 of 2023, 324,437 cases had been reported with an incidence of 33 per 100,000, 176,152 (55%) confirmed cases, 340 deaths with a fatality rate of 0.104, showing a 125% increase compared to the average of the last four years. The most affected countries were Paraguay, Brazil, Argentina, and Bolivia ^2^, while Zika cases during the same period were 27,397 cases with an incidence of 3 per 100,000, 2747 (10%) confirmed cases with a 22% increase compared to the average of the last 5 years, cases mainly registered in Brazil.

Another arbovirus that is reemerging in the Americas, especially in the Amazon region, is the Oropouche virus (OROV). In Brazil, as of epidemiological week 8 of 2024, OROV was detected in 2104 samples, with 1821 in Amazonas, 172 in Rondônia, 51 in Acre, and 12 in Roraima, all Brazilian states in the Amazon. Cases detected in 2023 and 2024 had a probable site of infection in states of the Northern region (Amazon) of Brazil (Acre, Amazonas, Pará, Rondônia, and Roraima), including cases reported in states of other regions of the country, in individuals who visited those states. In Peru during the same period of 2024, 146 cases of OROV were recorded in the departments of Loreto, Ucayali, and Madre de Dios, with the highest number of cases reported to date in this country. The provinces where confirmed cases were notified are: Maynas, Loreto (n=74); Coronel Portillo, Ucayali (n=38); Tambopata, Madre de Dios (n=14); Mariscal Ramón Castilla, Loreto (n=6); Tahuamanu, Madre de Dios (n=4); Atalaya, Ucayali (n=4); Padre Abad, Ucayali (n=4); and Datem del Marañón, Loreto (n=2) ^3^.

Another infection that has been overlooked in recent times is yellow fever; however, in Brazil, in 2016, the worst outbreak of sylvatic yellow fever (SYF) in the last 80 years began in the country. There was an exponential increase in the number of confirmed cases and deaths in humans, as well as epizootics in non-human primates (NHP), spreading from November 2016 to forested areas contiguous to the largest megalopolises in the Southeast region, such as São Paulo and Rio de Janeiro. This significantly increased the risk of urban reemergence of the disease, with cases extending into 2018. There are complex ecological-social determinants of this rapid viral spread of yellow fever, but also political constraints and inadequate strategies that have certainly contributed to accelerating and exacerbating this epidemiological scenario ^4^. Considering deforestation and the presence of *Aedes aegypti* in endemic yellow fever areas, the urban reemergence of yellow fever in the countries of the region awaits - like a sword of Damocles - a scenario that must be anticipated to ensure the availability of vaccines in our countries, which is feasible considering that currently only a single dose of the yellow fever vaccine is needed for lifelong protection.

These diseases caused by arboviruses not only affect the health of millions of people each year in Latin America but also generate a significant economic burden due to the necessary medical care and loss of productivity. The rapid spread and adaptability of vectors have amplified the vulnerability of our communities living in a mega-diverse region, experiencing the impact of climate change and global warming.

In this context, it is important to strengthen the capabilities that allow for early and accurate diagnosis of these diseases, which remains a challenge, since the symptoms often overlap and can be confused with other pathologies. A syndromic approach in the clinical aspect, supported by laboratory confirmation from the primary level of care, should be applied. Additionally, the lack of specific antiviral treatments underscores the urgent need for research and development of diagnostic tests for primary care and the search for effective antivirals.

Arboviral diseases not only threaten people’s health but also exert significant pressure on healthcare systems and local economies. The financial burden of medical care and the loss of human lives and productivity can lead to a vicious cycle of poverty and inequality, as is happening in Latin American countries.

**Figure 1:**
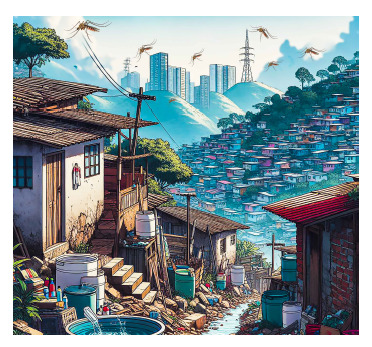
Social determinants of *Aedes aegypti*

Effectively addressing vector-borne diseases, especially those caused by arboviruses, requires a comprehensive approach that encompasses epidemiological surveillance, clinical management, prevention, vector control, and scientific research for the incorporation of new technologies with trained, organized, and available personnel both during epidemic and inter-epidemic periods, as well as the improvement of social determinants (Figure 1).

The search for innovative methods for vector control, such as residual spraying with a combination of insecticides, and the use of mosquitoes infected with Wolbachia, are control alternatives in the case of arboviruses transmitted by *Aedes aegypti*. However, it is also necessary to implement vector control strategies for other arboviruses such as Mayaro and Oropouche, which are on the rise and linked to deforestation. Likewise, the progressive inclusion of dengue vaccines, evaluating their safety and efficacy among the currently available options, and the search for vaccine prototypes against other arboviruses, are essential. Investment in technology and science is fundamental to addressing these challenges and protecting our communities from the threats of arboviruses, and it is even better if done collaboratively among countries in the Latin American region by exchanging and reducing technological gaps.

Effective education and communication are essential pillars in the fight against these diseases. Public awareness, promotion of preventive measures, and community participation are powerful tools for controlling the spread of arboviruses, emphasizing the need for qualitative research to achieve better results with the population’s acceptance of surveillance and control strategies.

Addressing arbovirus-caused diseases transmitted by vectors in the Americas requires a collective and coordinated effort. Governments, health institutions, the scientific community, industry, and society at large must develop robust and sustainable long-term strategies complementarily, to avoid repeating late and inadequate responses as countries experienced during the last COVID-19 pandemic.

Finally, it is imperative to reiterate that only through joint action and sustained investment in research, prevention, and treatment can we hope to overcome the current and future challenges posed by arboviruses in our Americas region. The health of our communities is at stake, and it is our responsibility to confront these threats with determination and solidarity, especially considering the intense migration, social determinants, and climate change that will inevitably accompany us in the medium and long term.
