# Supplementary material for: Growing threat of emerging and reemerging diseases: arboviruses and vector-borne diseases in the Americas
Source: Rev Peru Med Exp Salud Publica. 2024 Mar 27;41(1):4–6. doi: 10.17843/rpmesp.2024.411.13805 (PMC11149771; doi:10.17843/rpmesp.2024.411.13805)
Supplement: Translation

# Translation: Creciente amenaza de enfermedades emergentes y reemergentes: arbovirus y enfermedades transmitidas por vectores en las Américas

**Authors:** César Cabezas, Pedro F. C. Vasconcelos

**Affiliations:** 1 Instituto Nacional de Salud, Lima, Perú.; 2 Facultad de Medicina, Universidad Nacional Mayor de San Marcos, Lima, Perú.; 3 Departamento de Patologia, Universidade do Estado do Pará, Belém, Psará, Brasil.; 4 Seção de Arbovirologia e Febres Hemorrágicas, Instituto Evandro Chagas, Ananindeua, Pará, Brasil.

En las últimas décadas, las Américas han experimentado un incremento alarmante y sin precedentes de las enfermedades producidas por arbovirus, destacando la importancia crítica de abordar estos desafíos en el ámbito de la salud pública. Los arbovirus, acrónimo de la expresión virus transmitidos por artrópodos, son agentes virales transmitidos por vectores, y que han demostrado su capacidad para desencadenar epidemias devastadoras, afectando a comunidades enteras y presentando un riesgo significativo para la salud global de nuestros países y la región.

El dengue, Zika y chikungunya y la fiebre amarilla urbana, son enfermedades transmitidas por mosquitos del género *Aedes*, especialmente *Aedes aegypti*, que han emergido como protagonistas en la región de las Américas. En el caso del dengue, hasta la semana epidemiológica 8 del 2024, se reportaron un total de 1,874 021 casos sospechosos en las Américas, de los cuales 658 215 casos (35%) fueron confirmados por laboratorio y 1670 (0,1%) fueron clasificados como dengue grave, registrándose un total de 422 muertes por dengue, con una letalidad del 0,023% (incidencia acumulada de 205 casos por 100 000 habitantes). Esta cifra representa un incremento de 249% en comparación al mismo periodo del 2023 y 354% con respecto al promedio de los últimos 5 años ^1^.

Respecto al Chikunguya hasta la semana 30 del 2023 se habían registrado 324 437 casos con una incidencia de 33 x 100 000, 176 152 (55%) confirmados, 340 fallecidos con una letalidad de 0,104, mostrando un incremento de 125% en comparación con el promedio de los últimos cuatro años. Siendo los países más afectados Paraguay, Brasil, Argentina y Bolivia ^2^, mientras que los casos de Zika en el mismo periodo fueron 27 397 casos con una incidencia de 3 x 100 000, 2747 (10%) confirmados con un incremento del 22% en comparación con el promedio de los últimos 5 años, casos registrados principalmente en el Brasil ^2^.

Otro arbovirus que está reemergiendo en las Américas, sobre todo en la región amazónica es el virus de Oropuche (OROV), así en el Brasil, hasta la semana epidemiológica 8 del 2024, se detectó OROV en 2104 muestras, siendo 1821 en Amazonas, 172 en Rondônia, 51 en Acre y 12 en Roraima, todos estados brasileños de la Amazonia. Los casos detectados en 2023 y 2024 tuvieron un sitio probable de infección en estados de la región Norte (Amazonia) de Brasil (Acre, Amazonas, Pará, Rondônia y Roraima), incluyendo casos notificados en estados de otras regiones del país, en personas que visitaron esos estados ^3^. En el Perú en el mismo periodo del 2024, se registraron 146 casos de OROV en los departamentos de Loreto, Ucayali y Madre de Dios, siendo el mayor número de casos reportados a la fecha en este país. Las provincias en donde se notificaron los casos confirmados son: Maynas, Loreto (n=74); Coronel Portillo, Ucayali (n=38); Tambopata, Madre de Dios (n=14); Mariscal Ramón Castilla, Loreto (n=6); Tahuamanu, Madre de Dios (n=4); Atalaya, Ucayali (n=4); Padre Abad, Ucayali (n=4); y Datem del Marañón, Loreto (n=2) ^3^.

Otra infección poco visualizada en los últimos tiempos es la fiebre amarilla, sin embargo en el Brasil el año 2016 se inició el peor brote de fiebre amarilla silvestre (FAS) de los últimos 80 años en ese país, con un aumento exponencial en el número de casos confirmados y muertes en humanos y epizootias en primates no humanos (PNH), que se propagó desde noviembre de 2016 hacia áreas forestales contiguas a las mayores megalópolis del país en la región Sudeste, como São Paulo y Río de Janeiro, aumentando significativamente el riesgo de reurbanización de la enfermedad, con los casos que se extendieron hasta el 2018. Existen determinantes ecológico-sociales complejos de esta rápida propagación viral de la FA, pero también restricciones políticas y estrategias no adecuadas que ciertamente han contribuido a acelerar y agravar este escenario epidemiológico ^4^. Considerando la deforestación y la presencia del *Aedes aegypti* en zonas endémicas de fiebre amarilla aguarda -como una espada de Damócles- la reurbanización de la fiebre amarilla en los países de la región, hecho que debe ser previsto para contar con la disponibilidad de vacunas en nuestros países, lo cual es viable si tenemos en cuenta que actualmente sólo es necesario una sola dosis de la vacuna antiamarílica para proteger toda la vida.

Estas enfermedades causadas por los arbovirus no solo afectan la salud de millones de personas cada año en América Latina, sino que también generan una carga económica significativa debido a la atención médica necesaria y la pérdida de productividad. La propagación rápida y la adaptabilidad de los vectores han amplificado la vulnerabilidad de nuestras comunidades que viven en una región megadiversa, sufriendo el impacto del cambio climático y el calentamiento global.

En este contexto es importante fortalecer las capacidades que permitan un diagnóstico temprano y preciso de estas enfermedades lo cual sigue siendo un desafío, ya que los síntomas a menudo se superponen y pueden confundirse con otras patologías, debiendo aplicarse el enfoque sindrómico tanto en el aspecto clínico respaldado por la confirmación laboratorial desde el primer nivel de atención. Además, la falta de tratamientos antivirales específicos destaca la necesidad urgente de investigación y desarrollo tanto de pruebas de diagnóstico para el primer nivel de atención y la búsqueda de antivirales que resulten eficaces.

Las arbovirosis no solo amenazan la salud de las personas, sino que también ejercen una presión significativa sobre los sistemas de salud y las economías locales. La carga financiera de la atención médica y la pérdida de vidas humanas y de la productividad pueden llevar a un círculo vicioso de pobreza y desigualdad, como viene ocurriendo en los países latinoamericanos.

Enfrentar eficazmente las enfermedades transmitidas por vectores, en especial las producidas por arbovirus exige un enfoque integral que abarque la vigilancia epidemiológica, la clínica, la prevención, el control vectorial y la investigación científica para la incorporación de nuevas tecnologías con personal entrenado, organizado y disponible tanto en periodos epidémicos, como interepidémicos, así como la mejora de los determinantes sociales (Figura 1).

**Figura 1:** Determinantes sociales del *Aedes aegypti*

La búsqueda de métodos innovadores para el control vectorial, como la fumigación residual con combinación de insecticidas, así como el uso de mosquitos infectados con *Wolbachia* son alternativas de control en el caso de arbovirus transmitidos por el *Aedes aegypti*, sin embargo, también es necesario implementar estrategias de control vectorial en otras arbovirosis como el Mayaro y Oropuche que está en incremento y vinculados a la deforestación. Igualmente, la progresiva inclusión de vacunas contra el dengue evaluando en el camino su seguridad y eficacia de las actualmente disponibles, así como la búsqueda de prototipos de vacuna contra los otros arbovirus, son esenciales. La inversión en tecnología y ciencia es fundamental para enfrentar estos desafíos y proteger a nuestras comunidades de las amenazas de los arbovirus, y que mejor si lo hacemos colaborativamente entre los países de la región latinoamericana intercambiando y reduciendo las brechas tecnológicas.

La educación y la comunicación efectivas son pilares esenciales en la lucha contra estas enfermedades. La concienciación pública, la promoción de medidas preventivas y la participación comunitaria son herramientas poderosas para controlar la propagación de los arbovirus, debiendo destacar la necesidad de investigación cualitativa que permita mejores resultados con la aceptación de las estrategias de vigilancia y control, por parte de la población.

Enfrentar las enfermedades causadas por arbovirus y transmitidas por vectores en las Américas requiere un esfuerzo colectivo y coordinado. Es necesario que los gobiernos, las instituciones de salud, la comunidad científica, las organizaciones internacionales, la industria y la sociedad en general desarrollen complementariamente estrategias robustas y sostenibles a largo plazo, para no repetir respuestas tardías y no adecuadas como nos ocurrió a los países en la última pandemia del COVID-19.

Finalmente, es imperativo reiterar que solo a través de la acción conjunta y la inversión sostenida en investigación, prevención y tratamiento, podemos esperar superar los desafíos actuales y futuros planteados por los arbovirus en nuestra región de las Américas. La salud de nuestras comunidades está en juego, y es nuestra responsabilidad enfrentar estas amenazas con determinación y solidaridad, más aún considerando la intensa migración, las determinantes sociales y el cambio climático que nos acompañarán indefectiblemente en el mediano y largo plazo.
